# Draft Genomes of *Frankia* strains AiPa1 and AiPs1 Retrieved from Soil with Monocultures of *Picea abies* or *Pinus sylvestris* using *Alnus incana* as Capture Plant

**DOI:** 10.7150/jgen.77880

**Published:** 2023-01-01

**Authors:** Philippe Normand, Petar Pujic, Danis Abrouk, Spandana Vemulapally, Trina Guerra, Camila Carlos-Shanley, Dittmar Hahn

**Affiliations:** 1 Université Claude-Bernard Lyon 1, Université de Lyon, UMR 5557 CNRS Ecologie Microbienne, Villeurbanne, France.; 2 Texas State University, Department of Biology, 601 University Drive, San Marcos, TX 78666, USA.

**Keywords:** *Frankia*, Actinorhizal symbiosis, genome, nitrogen-fixing, biosynthetic gene clusters

## Abstract

The genomes of two nitrogen-fixing *Frankia* strains, AiPa1 and AiPs1, are described as representatives of two novel candidate species*.* Both strains were isolated from root nodules of *Alnus incana*, used as capture plants in bioassays on soils from a reforested site at Karttula, Finland, that was devoid of actinorhizal plants but contained 25 year-old monocultures of spruce (*Picea abies* (L.) Karsten) or pine (*Pinus sylvestris* L.), respectively. ANI analyses indicate that each strain represents a novel *Frankia* species, with genome sizes of 6.98 and 7.35 Mb for AiPa1 and AiPs1, respectively. Both genomes harbored genes typical for many other symbiotic frankiae, including genes essential for nitrogen-fixation, for synthesis of hopanoid lipids and iron-sulfur clusters, as well as clusters of orthologous genes, secondary metabolite determinants and transcriptional regulators. Genomes of AiPa1 and AiPs1 had lost 475 and 112 genes, respectively, compared to those of other cultivated *Alnus*-infective strains with large genomes. Lost genes included one *hup* cluster in AiPa1 and the *gvp* cluster in AiPs1, suggesting that some genome erosion has started to occur in a different manner in the two strains.

## Introduction

Technological advances in whole genome sequencing, in single-cell metagenomics and in comparative bioinformatics have revolutionized the description of microbial genera, species and subspecies 1-3. Comparative sequence analyses of whole genomes and the ANI metric 4 are now used as foundations for the classification of both cultured and uncultured microbes 5-7. Members of the genus *Frankia* are soil and nodule actinobacteria that have resisted isolation attempts for a long time. The first isolate was described only in 1978 8, after which many more followed 9-12. Differentiation of isolates has also been hampered by the limited availability of distinguishing phenotypic features between populations 13. Consequently, species of the genus *Frankia* have been scantily described for many years 14, 15.

Whole genome sequencing techniques have permitted to overcome these difficulties, resulting in the description of twelve species in the genus *Frankia* so far, with type strains deposited in international culture collections 16-18. Five candidate species have also been described using whole genome analyses of uncultured *Frankia* populations in root nodules 6, 18-20. The number of available whole genome sequences for *Frankia* strains has increased significantly during the last years, with many strains potentially representing new species 21-23. These data indicate that the genus *Frankia* is probably much more diverse than the twelve species and five candidate species described so far 13, 24-27. This statement is supported by recent genome analyses of *Frankia* strains isolated from nodules of *Alnus glutinosa* as representatives of three yet undescribed nitrogen-fixing symbiotic species 22, and by the identification of two additional species of non-nitrogen-fixing and non-symbiotic frankiae 21.

Comparative sequence analyses of amplicons of an actinobacteria-specific insertion in the 23S rRNA genes of frankiae identified strains AiPa1 and AiPs1 as additional candidates for the description of new species 28. Strains AiPa1 and AiPs1 have been isolated from root nodules of *Alnus incana* that was used as capture plant in bioassays aiming to determine the effects of 25 year-old monocultures of spruce (*Picea* abies (L.) Karsten) and pine (*Pinus sylvestris* L.) at a reforested site at Karttula, Finland (62° 53′, 26° 58′) on the nodulation capacity and diversity of frankiae in soils devoid of actinorhizal plants 29. Basic soil characteristics were virtually identical for both sites, characterized as a fine silty sand with 12 to 14% organic matter and a pH of 5.3 to 5.5 29, 30. The aim of this study was to use whole genome sequence analyses in order to evaluate and corroborate the potential of strains AiPa1 and AiPs1 for the description of new *Frankia* species.

## Materials and Methods

### Sample preparation

Defined Propionate Medium (DPM) containing propionate and NH_4_Cl as C and N source 31, respectively, was used to grow *Frankia* strains AiPa1 and AiPs1. Cells of both strains had been preserved in 20% v/v glycerol at -80°C since 2003. After two weeks of growth at 30°C, cells were harvested by centrifugation (15,000 x g, 5 min). After a brief sonication to disrupt aggregates of cell filaments (10 s at 20% output in a S-450 sonifier, Branson Ultrasonics, Danbury, CT) 32 followed by an additional centrifugation step, DNA was extracted from cell pellets using the SurePrep^TM^ Soil DNA Isolation Kit (Fisher Scientific, Houston, TX) 33. DNA was sent to the Microbial Genomics Sequencing Center, Pittsburgh, PA, USA, for library preparation and sequencing using standard protocols for the Illumina tagmentation and the NextSeq Illumina platform (2 x 150 bp).

### Genome assembly

Fastp was used with default settings to filter and trim sequence reads 34, where reads with average %GC<54 were removed using bbduk (https://jgi.doe.gov/data-and-tools/software-tools/bbtools/bb-tools-user-guide/). Genomes were assembled using SPAdes 3.13.0 35 and their quality checked with QUAST 36. Default values in the lineage workflow (lineage_set) in CheckM v1.0.18 were finally used to assess genome completeness and contamination 37.

### Comparative genomic analysis

We computed Average Nucleotide Identity (ANI) 4 of the assembled genomes of AiPa1 and AiPs1 with *Frankia* genomes of type strains of all described species and other selected genomes using the pyani platform with the b (Blast) setting (38; https://pyani.readthedocs.io). Clusters of orthologous genes (COGs) 39, secondary metabolite clusters and genes specific to or lost in the new genomes were identified through antiSMASH 40 on the Mage platform 41. An MLSA with AtpD, DnaA, FtsZ, Pgk, and RpoB was used to compute an AA distance matrix as done previously 42 to construct a phylogenetic tree using a rapid Neighbour Joining algorithm 43 and a bootstrap analysis 44.

## Results

### Characteristics of the two *Frankia* genomes

CheckM scores of 98.6% and 98.5% indicated that the genomes of strains AiPa1 and AiPs1, respectively, could be considered complete, while contamination indices of 0.27 and 4.05 demonstrated that they were pure. Genome sizes of AiPa1 and AiPs1 were 6.98 Mb and 7.35 Mb with GC contents of 71.12 and 72.13%, respectively, and were made up of 165 and 1,203 contigs with the largest contig being 310,535 and 128,115, respectively (Table 1).

### Phylogenetic analysis of *Frankia* spp

**Figure 1:**
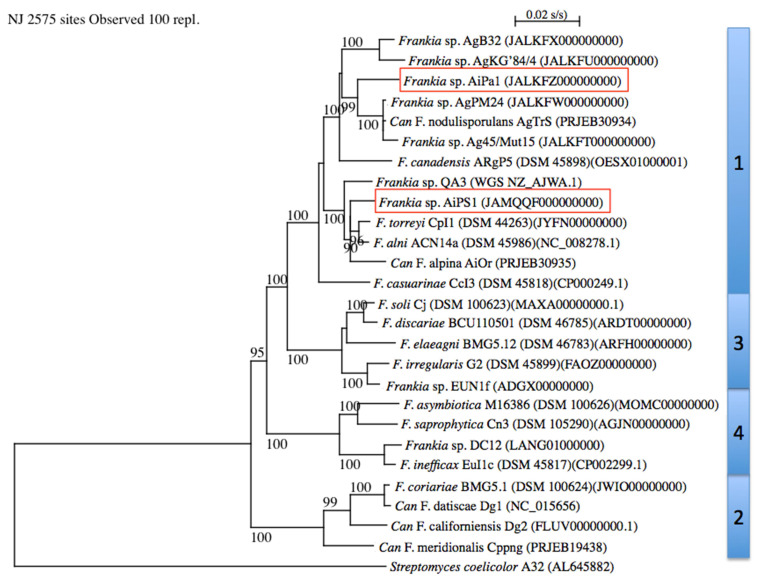
Phylogenetic tree of complete genomes using *Streptomyces coelicolor* A32 (AL645882) as outgroup. *Frankia* clusters are indicated on the right. Bootstrap results above 90% are given at nodes. The bar indicates 0.02%substitution/site. The two genomes described in the present study are framed.

**Figure 2:**
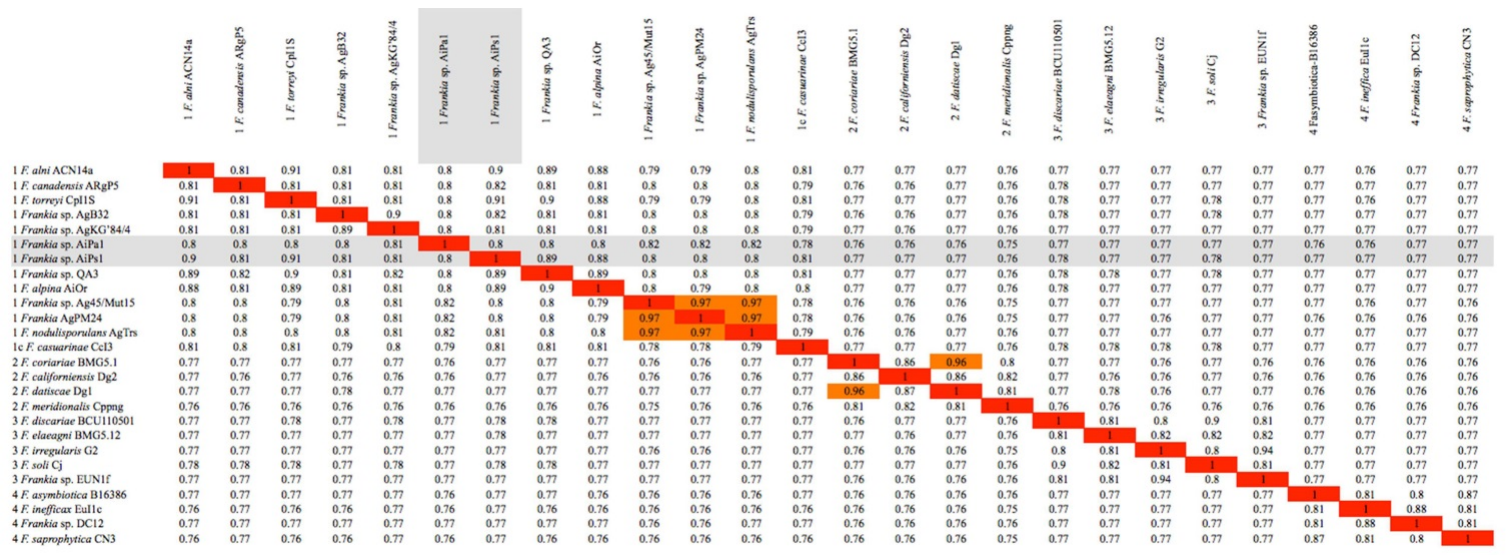
Heatmap matrix of Average Nucleotide Identity (ANI) comparisons for the *Frankia* genomes of strains and type strains of described species using the pyani platform with the b (Blast) setting 38; https://pyani.readthedocs.io). The two genomes described in the present study are highlighted in grey.

The MLSA with *Frankia* type strains revealed that strains AiPa1 and AiPs1 were members of *Frankia* cluster 1 (Figure 1). AiPa1 and AiPs1 represent two distinct lineages within the genus *Frankia*: strain AiPa1 has an ANI of 82% to its closest relatives (Ag45/Mut15 and AgPM24), with ANI percentages to other *Frankia* strains between 76 and 80%, while AiPs1 is closely related to, but distant from *Frankia alni* ACN14a and *Frankia torreyi* CpI1 with ANI values of 90 and 91%, well below the threshold of 95 proposed to delineate species (Figure 2). Both strains belong to cluster 1 frankiae, with ANI values ranging from 80% to 91%, while 76-77% values were obtained with cluster 2 genomes, and 77-78% with cluster 3 and 4 genomes (Figure 2).

### Analysis of functional genes in *Frankia* spp. isolates

Genes such as *nif, hup, suf, shc, cel, glx, bcsA* meant to be characteristic for the symbiosis were present in the genomes of symbiotic lineages (clusters 1, 2 and 3) compared to non-symbiotic lineages (cluster 4) (Table 1; Table S1). Both genomes lacked *gvp* genes encoding gas vesicle proteins, and one of the two *hup* clusters commonly found in cluster 1 frankiae was absent in AiPs1 but present in AiPa1.

Both the COG and the antiSMASH computation retrieved values for AiPa1 and AiPs1 characteristic of other* Alnus*-infective cluster 1 strains (Table 2 and 3). These data include high number of T1PKS and NRPS (Table 3) as well as transcriptional regulators such as ArsR, and LuxR (Table 4). Additional comparisons with cluster 2, 3 and 4 frankiae are provided in Supplemental tables S2, S3, and S4. A search for genes present in *F. alni* ACN14a, *Frankia* sp. QA3, *F. torreyi* CpI1 and *F. canadensis* ARgP5 but absent in AiPa1 and AiPs1 yielded 475 or 112 hits, respectively among which a *hup* cluster and a dipeptide transporter in AiPa1, and the *gvp* cluster in AiPs1 (Table S5).

## Discussion

Genome sizes of *Frankia* strains have been found to be quite variable, with size ranging from 4.9 Mb to 10.7 Mb. Initial studies determined relatively consistent sizes of 7.5 Mb to 7.7 Mb for genomes of *Alnus*-infective cluster 1 and some cluster 3 frankiae, however, other genomes of cluster 3 frankiae were much larger (9.0 to 10.4 Mb) 45-47, similar to many cluster 4 frankiae (8.8 to 10.7 Mb) 21, 48, 49. In contrast, genomes of cluster 1 species *Frankia casuarinae* (4.9 to 5.6 Mb) and Candidate species *F. nodulisporulans* (4.9 Mb) were smaller, similar to *F. coriariae* as cluster 2 representative (5.8 Mb) (see 50 for review).

Larger genomes have commonly been associated with duplications of genes involved in substrate transfers into central metabolic pathways 46. Strains with larger genomes might therefore be considered to have a higher potential to exploit a large variety of environments 20, 46. Smaller genomes like those of *F. casuarinae, F. nodulisporulans* and* F. coriariae* have been linked to genome reductions resulting in reduced saprotrophic potential, though their symbiotic potential is maintained 46. The genome sizes of 6.98 and 7.35 of strains AiPa1 and AiPs1 fit into recent discoveries of smaller genome sizes for many cluster 1 strains, with a size range from 6.4 Mb to 6.7 MB (Table 1). Smaller genome sizes coincide with the loss of some duplicated genes such as, for example, the *shc* gene coding for the synthesis of hopanoid lipids 46, or the *hup* genes coding for hydrogen uptake for the recycling of hydrogen derived from nitrogenase 51. Genes such as *hup* are lost by many strains (e.g. Ag45/Mut15, AgPM24, AgB32, AgKG'84/4) 22 including AiPa1, but not AiPs1 in this study, while others such as *shc* are lost less frequently (e.g. only in AgB32, AgKG'84/4) 23. The numbers of genes lost by individual strains differ significantly, e.g. 475 or 112 genes for AiPa1 and AiPs1, respectively, 380 or 409 genes for AgB32 and AgKG'84/4, or 459 genes for Ag45/Mut15 and AgPM24 22. Thus, genome reductions could be caused by genome erosion, which could be more pronounced in some strains such as Ag45/Mut15, AgPM24, AgB32 and AgKG'84/4 compared to others, including strains AiPa1 and AiPs1.

While smaller genomes have been found so far in frankiae with high symbiotic specificity and reduced saprophytic capabilities (e.g. *F. casuarinae, F. nodulisporulans* and* F. coriariae*), potential genome reduction is not indicative of reduced saprophytic growth in strains Ag45/Mut15, AgPM24 and AiPa1. Similar to species described for clusters 1 and 3, as well as for strains AgB32, AgKG'84/4 and AiPs1, all three strains grow in the rhizosphere of host and non-host plants, but in contrast to these species also proliferate in the presence of leaf litter as sole C- and N-sources 32, 52. Thus, these strains are able to grow on complex organic material such as leaf litter, and do not require easily available C-resources such as root exudates 53-56. Thus, genome erosions could have affected other traits of these strains, as indicated by the low competitive ability for nodule formation of strain Ag45/Mut15 compared to *F. torreyi* 57, 58.

Relatives of *Frankia* strain AiPa1 were exclusively or most prominently found in recently vegetated soils 59, 60, while relatives of AiPs1 were only found in the older part of the plantation (65 yrs), together with relatives of AiPa1, suggesting that environmental changes in time might have promoted *Frankia* population changes 59. Nodulation capacities of soils under spruce and pine monocultures were similar 61, with root nodule populations representing relatives of AiPa1 under spruce and AiPs1 under pine 29. However, both soils harbored other *Frankia* populations related to strain Ag45/Mut15 that is closely related to AiPa1 29, 61. Thus, it remains highly speculative to draw any conclusions about potential effects of plant species on the development of infective *Frankia* populations in soils under 25 year-old monocultures of spruce and pine.

The availability of genome information of strains AiPa1 and AiPs1 as representatives of two new species within cluster 1 of the genus *Frankia* provides the possibility to assess the importance of gene erosion, but also the presence of unique genes with respect to different physiological and potentially ecological adaptations. Thus, additional studies need to include analyses of unique and additional genes to highlight differences between these two strains and other representatives of cluster 1, and relate those differences to environmental characteristics.

## Supplementary Material

Supplementary tables.

## Figures and Tables

**Table 1:** Basic genome characteristics of *Frankia* strains AiPa1 and AiPs1 compared to those of strains and type strains of *Frankia* species in cluster 1. Additional comparisons with strains and type strains of clusters 2, 3 and 4 are provided in Supplemental Table S1.

	Cluster 1										
Strain	ACN14a^T^	ARgP5^T^	CpI1^T^	QA3	AgB32	AgKG'84/4	Ag45/Mut15	AgPM24	AiPa1	AiPs1	CcI3^T^
COG^1^	*alni*	*canadensis*	*torreyi*								*casuarinae*
G+C content (mol%)	72.8	72.4	72.4	72.6	72.22	72.13	71.37	71.35	71.12	72.13	70.1
Genome length (Mb)	7.50	7.73	7.62	7.59	6.71	6.51	6.44	6.67	6.98	7.35	5.43
# CDS	6,714	7,500	7,201	7,307	6,364	6,122	6,088	6,370	7,601	6,847	5,593
secondary metabolite clusters*	27	33	28	33	42	30	29	38	33	43	26
nifH**	1	1	1	1	1	1	1	1	1	1	1
*shc*	2	2	2	2	1	1	2	2	2	2	1
*hupL*	2	2	2	2	1	1	1	1	2	1	2
*sufD*	1	1	1	1	1	1	1	1	1	1	1
*celA1*	2	2	2	0	2	2	2	2	2	2	0
*glxA*	1	1	1	1	1	1	1	1	1	1	0
*bcsA*	1	1	1	0	1	1	1	1	1	1	0
*gvpJ*	1	1	1	0	0	0	0	0	0	0	0
*sodF*	1	1	1	1	1	1	1	1	1	1	1
*geoA*	1	1	1	1	1	1	1	1	1	1	1
*argG*	1	1	1	1	1	1	1	1	1	1	1
*accA*	1	1	1	1	1	1	1	1	1	1	1
*can*	2	2	2	2	2	2	2	2	1	2	2
*rhbE*	1	1	1	1	1	1	1	1	1	1	1
*lac*	1	1	1	1	1	1	1	1	1	1	1
*phdA*	1	1	1	1	1	1	1	1	1	1	1
*dctA*	1	1	1	1	1	1	1	1	1	0	1
*tgsA*	1	1	1	1	1	1	1	1	2	2	1
*ddnB*	1	1	1	1	1	0	1	1	0	2	0
*mopB*	1	1	2	2	2	2	2	2	2	2	1
*qorB*	1	2	1	1	1	0	0	0	2	1	0
*glbN*	1	1	1	2	1	1	1	1	2	2	1
# contigs	1	568	153	120	274	342	157	230	165	1,203	1
Accession	NC_008278.1	OESX01000001	JYFN00000000	WGS NZ_AJWA.1	JALKFX000000000	JALKFU000000000	JALKFT000000000	JALKFW000000000	JALKFW000000000	JAMQQF000000000	CP000249.1
Reference	(46)	(62)	(63)	(64)	(23)	(23)	(22)	(22)	This study	This study	(46)

* indicates the number of clusters identified by AntiSMASH ** indicates the number of hits (>50%) following a BlastP. *nif* is nitrogenase, *shc* is squalene hopene cyclase, *hup* is hydrogenase uptake, *suf* is sulfur-iron cluster, *cel* is cellulase, *glx* is glucose oxidase, *bcs* is cellulose synthase, *gvp* is gas vesicle cluster, *sodF* is superoxide dismutase iron, *geoA* is geosmine synthase, *arG* is arginine, *acc* is acetate carboxylase, *can* is carbonic anhydrase, *rhb* is rhizobactin, *lac* is laccase, *phd* is a phytoene desaturase, *dct* is a dicarboxylate transporter, *tgs* is diacylglycerol O-acyltransferase. *ddn* is F_420_H(2)-dependent quinone nitroreductase, *mop* is molybdenum transport, *qor* is quinone oxydoreductase, *glb* is hemoglobin.

**Table 2:** COG characteristics of *Frankia* strains AiPa1 and AiPs1 compared to those of type strains and strains of *Frankia* species in cluster 1. Additional comparisons with strains and type strains of clusters 2, 3 and 4 are provided in Supplemental Table S2.

	Cluster 1										
Strain	ACN14a^T^	ARgP5^T^	CpI1^T^	QA3	AgB32	AgKG'84/4	Ag45/Mut15	AgPM24	AiPa1	AiPs1	CcI3^T^
COG^1^	*alni*	*canadensis*	*torreyi*								*casuarinae*
D	56	66	75	64	64	51	56	61	63	63	57
M	241	189	253	236	216	212	225	241	221	289	207
N	19	15	26	22	17	13	12	17	21	16	12
O	181	134	181	190	143	150	133	140	158	184	147
T	325	226	320	326	318	281	291	290	292	327	232
U	42	38	50	38	45	54	45	50	46	47	48
V	94	74	86	102	83	76	77	81	83	80	60
J	212	226	212	257	222	219	209	212	218	253	202
K	565	402	594	646	537	507	509	525	534	610	369
L	270	254	351	356	332	266	308	319	289	306	433
C	435	323	455	472	348	355	346	347	376	458	256
E	523	386	482	534	440	463	452	451	458	534	335
F	111	82	104	108	91	100	96	94	98	105	94
G	326	274	321	342	307	298	289	297	286	352	233
H	192	149	186	187	181	186	170	184	166	186	174
I	432	258	400	460	299	331	296	303	328	489	191
P	311	243	323	332	274	293	307	313	274	299	210
Q	376	226	368	371	323	331	304	339	330	449	197
R	1009	704	1005	1059	863	869	814	836	893	1055	619
S	301	226	315	286	281	268	258	278	266	288	223

^1^class: **D**: Cell cycle control, cell division, chromosome partitioning; **M**: Cell wall/membrane/envelope biogenesis; **N**: Cell motility; **O**: Posttranslational modification, protein turnover, chaperones; **T**: Signal transduction mechanisms; **U**: Intracellular trafficking, secretion, and vesicular transport; **V**: Defense mechanisms; **J**: Translation, ribosomal structure and biogenesis; **K**: Transcription; **L**: Replication, recombination and repair; **C**: Energy production and conversion; **E**: Amino acid transport and metabolism; **F**: Nucleotide transport and metabolism; **G**: Carbohydrate transport and metabolism; **H**: Coenzyme transport and metabolism; **I**: Lipid transport and metabolism; **P**: Inorganic ion transport and metabolism; **Q**: Secondary metabolites biosynthesis, transport and catabolism; **R**: General function prediction only; **S**: Function unknown.

**Table 3:** Number of secondary metabolites clusters (antiSMASH) of *Frankia* strains AiPa1 and AiPs1 compared to those of cultivated strains and type strains of *Frankia* species in cluster 1. Additional comparisons with strains and type strains of clusters 2, 3 and 4 are provided in Supplemental Table S3.

	Cluster 1										
Strain	ACN14a^T^	ARgP5^T^	CpI1^T^	QA3	AgB32	AgKG'84/4	Ag45/Mut15	AgPM24	AiPa1	AiPs1	CcI3^T^
	*alni*	*canadensis*	*torreyi*								*casuarinae*
t1PKS^1^	6	9	8	8	10	13	9	11	9	10	1
t2PKS	1	3	1	3	2	1	1	1	1	2	2
t3PKS	1	1	1	1	1	1	1	1	1	1	0
otherKS	4	4	3	3	5	4	3	5	4	3	4
t1pks-NRPS	1	0	1	0	1	0	1	2	0	0	1
NRPS	3	6	2	2	6	8	6	6	4	8	0
terpene	5	3	5	5	3	6	4	4	5	6	4
lanthipeptide	1	1	1	3	2	1	0	3	3	0	6
bacteriocin	2	1	2	2	2	3	1	2	1	1	1
siderophore	1	1	1	1	1	2	1	1	2	1	1
lassopeptide	1	1	1	2	1	1	1	1	1	1	0
betalactone	0	0	0	0	0	0	0	0	3	0	0
thiopeptide	0	0	0	0	0	0	0	0	0	0	1
butyrolactone	0	0	0	0	0	0	0	0	0	0	0
phosphonate	1	1	0	0	1	0	0	0	0	0	0
arylpolyene	0	0	0	0	0	0	0	0	0	0	0
nucleoside	0	0	0	0	0	0	0	0	0	0	1
ladderane	0	0	0	0	0	0	0	0	0	0	0
oligosaccharide	0	0	0	0	0	0	0	0	0	0	0
resorcinol	0	0	0	0	0	0	0	0	0	0	0
LAP	0	0	0	0	1	0	0	1	0	0	0
other	0	2	2	3	2	3	1	0	2	0	4
											
Total/strain	27	33	28	33	38	43	29	38	36	33	26

^1^tnPKS is type "n" Polyketide Synthase, NRPS is Non Ribosomal Peptide Synthase, LAP is Linear Azole/azoline-containing Peptide.

**Table 4:** Number of transcriptional regulators of *Frankia* strains AiPa1 and AiPs1 compared to those of strains and type strains of *Frankia* species in cluster 1. Additional comparisons with strains and type strains of clusters 2, 3 and 4 are provided in Supplemental Table S4.

	Cluster 1										
Strain	ACN14a^T^	ARgP5^T^	CpI1^T^	QA3	AgB32	AgKG'84/4	Ag45/Mut15	AgPM24	AiPa1	AiPs1	CcI3^T^
Class^1^	*alni*	*canadensis*	*torreyi*								*casuarinae*
AraC	9	9	10	16	9	13	6	6	12	16	2
ArsR	9	6	5	1	14	13	7	6	22	15	6
AsnC	3	2	2	4	3	4	4	3	4	4	3
CRP	4	2	1	1	4	3	4	4	5	6	2
DeoR	4	1	0	0	4	4	1	2	6	7	0
DtxR	1	1	1	1	1	1	1	1	1	1	1
FurC	2	3	3	4	3	3	3	3	3	4	2
GntR	25	19	10	20	12	15	7	5	22	20	6
IclR	3	6	4	9	7	8	4	3	12	5	2
LuxR	10	19	19	36	40	36	10	14	37	29	20
LysR	18	16	12	22	18	18	11	10	28	25	5
MarR	21	19	13	33	23	20	16	15	34	27	15
MerR	8	17	9	22	19	18	10	10	25	16	12

^1^class: **AraC**: arabinose regulator; **ArsR**: arsenic resistance; **AsnC**: asparagine synthase regulator; **CRP**: cyclic AMP receptor protein (catabolite repression); **DeoR**: deoxyribonucleoside synthesis operon regulator; **DtxR**: diphtheria toxin repressor; **FurC**: ferric uptake regulator; **GntR**: gluconate regulator; **IclR**: isocitrate lyase regulator; **LuxR**: quorum-sensing luminescence regulator; **LysR**: lysine regulator; **MarR**: Multiple antibiotic resistance regulator; **MerR**: mercury resistance regulator; **TetR**: Tetracycline repressor; **WhiB**: regulation of morphological differentiation.

## References

[1] Vollmers J, Wiegand S, Kaster AK (2017). Comparing and evaluating metagenome assembly tools from a microbiologist's perspective - not only size matters!. PLoS One.

[2] Tambong JT, Xu RL, Cuppels D, Chapados J, Gerdis S, Eyres J (2022). Whole-genome resources and species-level taxonomic validation of 89 plant-pathogenic *Xanthomonas* strains isolated from various host plants. Plant Dis.

[3] Zareba-Marchewka K, Szymanska-Czerwinska M, Livingstone M, Longbottom D, Niemczuk K (2021). Whole genome sequencing and comparative genome analyses of *Chlamydia abortus* strains of avian origin suggests that *Chlamydia abortus* species should be expanded to include avian and mammalian subgroups. Pathogens.

[4] Goris J, Konstantinidis KT, Klappenbach JA, Coenye T, Vandamme P, Tiedje JM (2007). DNA-DNA hybridization values and their relationship to whole-genome sequence similarities. Int J Syst Evol Microbiol.

[5] Gilroy R, Leng J, Ravi A, Adriaenssens EM, Oren A, Baker D (2022). Metagenomic investigation of the equine faecal microbiome reveals extensive taxonomic diversity. Peerj.

[6] Nguyen TV, Wibberg D, Vigil-Stenman T, Berckx F, Battenberg K, Demchenko KN (2019). *Frankia*-enriched metagenomes from the earliest diverging symbiotic *Frankia* cluster: they come in teams. Genome Biol Evol.

[7] Nguyen HDT, Dodge A, Dadej K, Rintoul TL, Ponomareva E, Martin FN (2022). Whole genome sequencing and phylogenomic analysis show support for the splitting of g genus *Pythium*. Mycologia.

[8] Callaham D, Del Tredici P, Torrey JG (1978). Isolation and cultivation *in vitro* of the actinomycete causing root nodulation in *Comptonia*. Science.

[9] Benson DR (1982). Isolation of *Frankia* strains from alder actinorhizal root nodules. Appl Environ Microbiol.

[10] Caru M, Becerra A, Sepulveda D, Cabello A (2000). Isolation of infective and effective *Frankia* strains from root nodules of *Alnus acuminata* (Betulaceae). World J Microb Biot.

[11] Gtari M, Brusetti L, Skander G, Mora D, Boudabous A, Daffonchio D (2004). Isolation of *Elaeagnus*-compatible *Frankia* from soils collected in Tunisia. FEMS Microbiol Lett.

[12] Gtari M, Ghodhbane-Gtari F, Nouioui I, Ktari A, Hezbri K, Mimouni W (2015). Cultivating the uncultured: growing the recalcitrant cluster-2 *Frankia* strains. Sci Rep-Uk.

[13] Fernandez MP, Meugnier H, Grimont PAD, Bardin R (1989). Deoxyribonucleic acid relatedness among members of the genus *Frankia*. Int J Syst Bacteriol.

[14] Lechevalier MP (1994). Taxonomy of the genus *Frankia* (Actinomycetales). Int J Syst Bacteriol.

[15] Lechevalier MP, Lechevalier HA (1990). Systematics, isolation, and culture of *Frankia*. In: Schwintzer C.R, Tjepkema J.D. (Eds.) The biology of *Frankia* and actinorhizal plants, Academic Press, New York.

[16] Normand P, Queiroux C, Tisa LS, Benson DR, Rouy Z, Cruveiller S (2007). Exploring the genomes of *Frankia*. Physiol Plantarum.

[17] Tisa LS, Oshone R, Sarkar I, Ktari A, Sen A, Gtari M (2016). Genomic approaches toward understanding the actinorhizal symbiosis: an update on the status of the *Frankia* genomes. Symbiosis.

[18] Normand P, Fernandez MP (2019). *Frankia* Brunchorst 1886, 174^AL^. In: Whitman W.B, Rainey F.A, Kämpfer P, Trujillo M.E, DeVos P, Hedlund B, Dedysh S. (Eds. ) Bergey's Manual of Systematics of Archaea and Bacteria.

[19] Bethencourt L, Vautrin F, Taib N, Dubost A, Castro-Garcia L, Imbaud O (2019). Draft genome sequences for three unisolated *Alnus*-infective *Frankia* Sp+ strains, AgTrS, AiOr and AvVan, the first sequenced *Frankia* strains able to sporulate *in-planta*. J. Genomics.

[20] Persson T, Battenberg K, Demina IV, Vigil-Stenman T, Heuvel BV, Pujic P (2015). Candidatus *Frankia datiscae* Dg1, the actinobacterial microsymbiont of *Datisca glomerata*, expresses the canonical nod genes nodABC in symbiosis with its host plant. PLoS One.

[21] Carlos-Shanley C, Guerra T, Hahn D (2021). Draft genomes of non-nitrogen-fixing *Frankia* strains. J Genomics.

[22] Normand P, Pujic P, Abrouk D, Vemulapally S, Guerra T, Carlos-Shanley C (2022). Draft genomes of nitrogen-fixing *Frankia* strains Ag45/Mut15 and AgPM24 isolated from root nodules of *Alnus glutinosa*. J Genomics.

[23] Normand P, Pujic P, Abrouk D, Vemulapally S, Guerra T, Carlos-Shanley C (2022). Draft genomes of symbiotic *Frankia* strains AgB32 and AgKG'84/4 from root nodules of *Alnus glutinosa* growing under contrasted environmental conditions. J Genomics.

[25] Hahn D, Mirza B, Benagli C, Vogel G, Tonolla M (2011). Typing of nitrogen-fixing *Frankia* strains by matrix-assisted laser desorption ionization-time-of-flight (MALDI-TOF) mass spectrometry. Syst Appl Microbiol.

[26] Pozzi AC, Bautista-Guerrero HH, Abby SS, Herrera-Belaroussi A, Abrouk D, Normand P (2018). Robust *Frankia* phylogeny, species delineation and intraspeciesdiversity based on Multi-Locus Sequence Analysis (MLSA) and Single-Locus Strain Typing (SLST) adapted to a large sample size. Syst Appl Microbiol.

[27] Normand P, Orso S, Cournoyer B, Jeannin P, Chapelon C, Dawson J (1996). Molecular phylogeny of the genus *Frankia* and related genera and emendation of the family Frankiaceae. Int J Syst Bacteriol.

[28] Ben Tekaya S, Ganesan AS, Guerra T, Dawson JO, Forstner MRJ, Hahn D (2017). SybrGreen- and TaqMan-based quantitative PCR approaches allow assessment of the abundance and relative distribution of *Frankia* clusters in soils. Appl Environ Microb.

[29] Maunuksela L, Zepp K, Koivula T, Zeyer J, Haahtela K, Hahn D (1999). Analysis of *Frankia* populations in three soils devoid of actinorhizal plants. FEMS Microbiol Ecol.

[30] Priha O, Smolander A (1997). Microbial biomass and activity in soil and litter under *Pinus sylvestris*, *Picea abies* and *Betula pendula* at originally similar field afforestation sites. Biol Fertil Soils.

[31] Meesters TM, van Genesen ST, Akkermans ADL (1985). Growth, acetylene reduction activity and localization of nitrogenase in relation to vesicle formation in *Frankia* strains Cc1.17 and Cp1.2. Arch Microbiol.

[32] Mirza BS, Welsh A, Hahn D (2007). Saprophytic growth of inoculated *Frankia* sp. in soil microcosms. FEMS Microbiol Ecol.

[33] Samant S, Sha Q, Iyer A, Dhabekar P, Hahn D (2012). Quantification of *Frankia* in soils using SYBR Green based qPCR. Syst Appl Microbiol.

[34] Chen S, Zhou Y, Chen Y, Gu J (2018). fastp: an ultra-fast all-in-one FASTQ preprocessor. Bioinformatics.

[35] Prjibelski A, Antipov D, Meleshko D, Lapidus A, Korobeynikov A (2020). Using SPAdes *de novo* assembler. Curr Protoc Bioinformatics.

[36] Gurevich A, Saveliev V, Vyahhi N, Tesler G (2013). QUAST: quality assessment tool for genome assemblies. Bioinformatics.

[37] Parks DH, Imelfort M, Skennerton CT, Hugenholtz P, Tyson GW (2015). CheckM: assessing the quality of microbial genomes recovered from isolates, single cells, and metagenomes. Genome Res.

[38] Pritchard L, Glover RH, Humphris S, Elphinstone JG, Toth IK (2016). Genomics and taxonomy in diagnostics for food security: soft-rotting enterobacterial plant pathogens. Anal Methods-Uk.

[39] Tatusov RL, Natale DA, Garkavtsev IV, Tatusova TA, Shankavaram UT, Rao BS (2001). The COG database: new developments in phylogenetic classification of proteins from complete genomes. Nucleic Acids Res.

[40] Medema MH, Blin K, Cimermancic P, de Jager V, Zakrzewski P, Fischbach MA (2011). antiSMASH: rapid identification, annotation and analysis of secondary metabolite biosynthesis gene clusters in bacterial and fungal genome sequences. Nucleic Acids Res.

[41] Vallenet D, Calteau A, Cruveiller S, Gachet M, Lajus A, Josso A (2017). MicroScope in 2017: an expanding and evolving integrated resource for community expertise of microbial genomes. Nucleic Acids Res.

[42] Ondov BD, Treangen TJ, Melsted P, Mallonee AB, Bergman NH, Koren S (2016). Mash: fast genome and metagenome distance estimation using MinHash. Genome Biol.

[43] Simonsen M, Mailund T, Pedersen CNS (2008). Rapid Neighbour-Joining. In: Crandall K.A, Lagergren J. (Eds.) WABI 2008: Algorithms in Bioinformatics, Springer Verlag, Heidelberg.

[44] Felsenstein J (1985). Confidence limits of phylogenies: an approach using the bootstrap. Evolution.

[45] Pujic P, Bolotin A, Fournier P, Sorokin A, Lapidus A, Richau KH (2015). Genome sequence of the atypical symbiotic *Frankia* R43 strain, a nitrogen-fixing and hydrogen-producing actinobacterium. Genome Announc.

[46] Normand P, Lapierre P, Tisa LS, Gogarten JP, Alloisio N, Bagnarol E (2007). Genome characteristics of facultatively symbiotic *Frankia* sp strains reflect host range and host plant biogeography. Genome Res.

[47] Nouioui I, Ghodhbane-Gtari F, Rhode M, Sangal V, Klenk HP, Gtari M (2018). *Frankia irregularis* sp nov, an actinobacterium unable to nodulate its original host, *Casuarina equisetifolia*, but effectively nodulates members of the actinorhizal Rhamnales. Int J System Evol Microbiol.

[48] Ghodhbane-Gtari F, Beauchemin N, Bruce D, Chain P, Chen A, Walston Davenport K (2013). Draft genome sequence of *Frankia* sp. strain CN3, an atypical, noninfective (Nod-) ineffective (Fix-) isolate from *Coriaria nepalensis*. Genome Announc.

[49] Nouioui I, Gueddou A, Ghodhbane-Gtari F, Rhode M, Gtari M, Klenk HP (2017). *Frankia asymbiotica* sp nov, a non-infective actinobacterium isolated from *Morella californica* root nodule. Int J Syst Evol Micr.

[50] Gtari M, Nouioui I, Sarkar I, Ghodhbane-Gtari F, Tisa LS, Sen A (2019). An update on the taxonomy of the genus *Frankia* Brunchorst, 1886, 174^AL^. Anton Leeuw.

[51] Richau KH, Kudahettige RL, Pujic P, Kudahettige NP, Sellstedt A (2013). Structural and gene expression analyses of uptake hydrogenases and other proteins involved in nitrogenase protection in *Frankia*. J Biosciences.

[52] Mirza BS, Welsh AK, Hahn D (2009). Growth of *Frankia* strains in leaf litter-amended soil and the rhizosphere of a non-actinorhizal plant. FEMS Microbiol. Ecol.

[53] Smolander A (1990). *Frankia* populations in soils under different tree species - with special emphasis on soils under *Betula pendula*. Plant Soil.

[54] Rönkkö R, Smolander A, Nurmiaho-Lassila EL, Haahtela K (1993). *Frankia* in the rhizosphere of nonhost plants: A comparison with root-associated nitrogen-fixing *Enterobacter*, *Klebsiella* and *Pseudomonas*. Plant Soil.

[55] Nickel A, Pelz O, Hahn D, Saurer M, Siegwolf R, Zeyer J (2001). Effect of inoculation and leaf litter amendment on establishment of nodule-forming *Frankia* populations in soil. Appl Environ Microbiol.

[56] Samant S, Dawson JO, Hahn D (2016). Growth responses of introduced *Frankia* strains to edaphic factors. Plant Soil.

[57] Vemulapally S, Guerra T, Hahn D (2022). Effect of different *Alnus* taxa on abundance and diversity of introduced and indigenous *Frankia* in soils and root nodules. FEMS Microbiol Ecol.

[58] Vemulapally S, Guerra T, Weckerly FW, Hahn D (2022). Competition of two inoculated *Frankia* strains in root nodulation of *Alnus glutinosa* seedlings and associated *Frankia*-strain growth in rhizospheric and non-rhizospheric soils. Plant Soil.

[59] Samant S, Amann RI, Hahn D (2014). Evaluation of the 23S rRNA gene as target for qPCR based quantification of *Frankia* in soils. Syst Appl Microbiol.

[60] Samant S, Sha Q, Iyer A, Dhabekar P, Hahn D (2012). Quantification of *Frankia* in soils using SYBR Green based qPCR. Syst Appl Microbiol.

[61] Maunuksela L, Hahn D, Haahtela K (2000). Effect of freezing of soils on nodulation capacities of total and specific *Frankia* populations. Symbiosis.

[62] Normand P, Nouioui I, Pujic P, Fournier P, Dubost A, Schwob G (2018). *Frankia canadensis* sp nov, isolated from root nodules of *Alnus incana* subspecies *rugosa*. Int J Syst Evol Microbiol.

[63] Oshone R, Hurst SGt, Abebe-Akele F, Simpson S, Morris K, Thomas WK (2016). Permanent draft genome sequences for two variants of *Frankia* sp. strain CpI1, the first *Frankia* strain isolated from root nodules of *Comptonia peregrina*. Genome Announc.

[64] Sen A, Beauchemin N, Bruce D, Chain P, Chen A, Walston Davenport K (2013). Draft genome sequence of *Frankia* sp. strain QA3, a nitrogen-fixing actinobacterium isolated from the root nodule of *Alnus nitida*. Genome Announc.

[65] Wall LG, Beauchemin N, Cantor MN, Chaia E, Chen A, Detter JC (2013). Draft genome sequence of *Frankia* sp. strain BCU110501, a nitrogen-fixing actinobacterium isolated from nodules of *Discaria trinevis*. Genome Announc.

[66] Nouioui I, Beauchemin N, Cantor MN, Chen A, Detter JC, Furnholm T (2013). Draft genome sequence of *Frankia* sp. strain BMG5.12, a nitrogen-fixing actinobacterium isolated from Tunisian soils. Genome Announc.

[67] Nouioui I, Gtari M, Goker M, Ghodhbane-Gtari F, Tisa LS, Fernandez MP (2016). Draft genome sequence of *Frankia* strain G2, a nitrogen-fixing actinobacterium isolated from *Casuarina equisetifolia* and able to nodulate actinorhizal plants of the order Rhamnales. Genome Announc.

[68] Gtari M, Ghodhbane-Gtari F, Nouioui I (2020). *Frankia soli* sp. nov, an actinobacterium isolated from soil beneath *Ceanothus jepsonii*. Int J Syst Evol Microbiol.

[69] Tisa LS, Beauchemin N, Cantor MN, Furnholm T, Ghodhbane-Gtari F, Goodwin L (2015). Draft genome sequence of *Frankia* sp. strain DC12, an atypical, noninfective, ineffective isolate from *Datisca cannabina*. Genome Announc.

